# Acceptance of and adherence with long-term positive airway pressure treatment in adults with chronic obstructive pulmonary disease: A systematic review protocol

**DOI:** 10.1371/journal.pone.0287887

**Published:** 2023-07-03

**Authors:** Cheryl R. Laratta, Linn E. Moore, Rachel Jen, Sandra M. Campbell, Joanna E. MacLean, Sachin R. Pendharkar, Brian H. Rowe

**Affiliations:** 1 Department of Medicine, University of Alberta, Edmonton, Alberta, Canada; 2 School of Public Health, University of Alberta, Edmonton, Alberta, Canada; 3 Department of Pediatrics, University of Alberta, Edmonton, Alberta, Canada; 4 Department of Medicine, University of British Columbia, Vancouver, British Columbia, Canada; 5 John W. Scott Health Sciences Library, University of Alberta, Edmonton, Alberta, Canada; 6 Department of Medicine, Cumming School of Medicine, University of Calgary, Calgary, Alberta, Canada; 7 Department of Community Health Sciences, Cumming School of Medicine, University of Calgary, Calgary, Alberta, Canada; 8 Department of Emergency Medicine, University of Alberta, Edmonton, Alberta, Canada; University of Toronto, CANADA

## Abstract

**Background:**

Long-term noninvasive positive airway pressure (PAP) treatment is effective treatment for sleep-related breathing disorders and chronic hypercarbic respiratory failure secondary to chronic obstructive pulmonary disease (COPD). PAP treatment may be delivered as continuous positive airway pressure or noninvasive ventilation. Success in initiating PAP treatment and barriers to its use in adult patients with COPD are largely unknown. This systematic review aims to identify the acceptance of and adherence to PAP treatment prescribed for long-term use in adult patients with COPD and to summarize variables associated with these measures.

**Methods:**

Seven online electronic databases will be searched by an experienced medical librarian to identify records containing the concepts “obstructive airways disease” and “noninvasive positive airway pressure” and “acceptance” or “adherence”. Randomized and non-randomized studies of interventions will be included. Citation lists from relevant articles will be reviewed, and experts will be contacted regarding unpublished studies. Abstracts from key conferences between 2018–2023 and Google Scholar search results will be reviewed for inclusion. Titles, abstracts and full texts will be reviewed independently for inclusion by two reviewers. Data extraction will be completed by one author using a pre-established form and primary outcomes confirmed by a second author. Methodological quality will be evaluated. If sufficient data are available for meta-analysis, a pooled summary statistic for the primary outcome will be calculated using a random-effects generic inverse-variance meta-analysis, weighted proportion or weighted medians-based approach. Subgroup analysis will explore clinically meaningful sources of heterogeneity. Variables that are associated with acceptance and adherence will be described.

**Discussion:**

Long-term PAP treatment is a complex intervention prescribed to patients with COPD for several indications. Synthesis of the evidence on success with PAP treatment and variables associated with acceptance or adherence will inform program and policy development for supporting patients with COPD who are prescribed this therapy.

**Trial registration:**

**Systematic review registration:** This protocol was registered with the International Prospective Register of Systematic Reviews (PROSPERO) on July 13, 2021 (registration number CRD42021259262), with revisions submitted on April 17, 2023.

## Introduction

Chronic obstructive pulmonary disease (COPD) is a complex, multifaceted disease that may be complicated by respiratory failure and comorbidities such as sleep related breathing disorders that contribute to poor health outcomes [1]. Hypercarbic respiratory failure attributed to COPD is associated with high mortality and a poor quality of life [2]. Obstructive sleep apnea (OSA), the most common sleep-related breathing disorder, is associated with lower health-related quality of life [3] and increased health care utilization [4] in patients with COPD, and may co-exist with chronic hypercarbic respiratory failure [5]. These conditions share a common treatment of positive airway pressure (PAP). While the context within which PAP treatment is prescribed varies among patients with COPD, barriers to success with PAP therapy may be similar across disease indications.

Noninvasive PAP treatment intended for long-term use, generally during sleep, may be delivered as continuous positive airway pressure (CPAP) or noninvasive ventilation (NIV). CPAP is an established therapy for OSA and has been shown to improve quality of life and reduce daytime sleepiness [6], with similar results to NIV [7, 8]. In patients with COPD, observational studies suggest that CPAP treatment for OSA is associated with longer survival [9, 10], longer time to first exacerbation requiring hospitalization [11], fewer Emergency Department (ED) visits [12], and fewer hospitalizations from COPD exacerbations [12, 13]. For sleep related breathing disorders, NIV may be prescribed when CPAP does not achieve physiologic treatment targets [14, 15], to resolve CPAP intolerance [16, 17], or at discharge from hospital for patients with obesity and chronic hypercapnic respiratory failure [18]. NIV is also indicated to treat chronic hypercarbic respiratory failure secondary to COPD as randomized controlled trials support that NIV prevents hospital readmission and intubation as well as improves survival [19], particularly when high intensity NIV is prescribed [20]. Evidence supporting the use of CPAP for chronic hypercapnic respiratory failure is primarily derived in patients with obesity without significant airflow obstruction where CPAP and NIV have similar long-term benefits [21]. Despite the proven benefits, PAP treatment is a complex and challenging intervention to accept and use [22]. It is therefore critically important that as the indications for PAP treatment in patients with COPD expand, so does our understanding of how to help patients be successful with therapy.

The aim of this review is to characterize the acceptance of and adherence to long-term noninvasive PAP treatment in adult patients with COPD. The World Health Organization identifies broad categories of barriers to intervention adherence [23]. In the context of CPAP, factors that represent challenges to adherence in the general population of patients with OSA are classified as disease characteristics, sociodemographic characteristics, psychosocial factors, treatment titration procedure, technological device factors and side effects [24, 25]. There are, however, contextual factors among patients with COPD that may result in different barriers to PAP adherence compared to other patient populations. These factors include health behaviours, health literacy, baseline symptoms and contact with specialists. Through subgroup analysis of the primary aim, we will explore whether pre-specified variables such as specific disease characteristics or aspects of health service delivery are associated with either acceptance or adherence. The secondary aim is to summarize known variables that are associated with either acceptance or adherence in this population. The results of this review will be used to inform programs and policies designed to support patients with COPD who are prescribed PAP treatment and identify gaps in the literature that need to be addressed through future research.

## Materials and methods

### Study design

**Fig 1:**
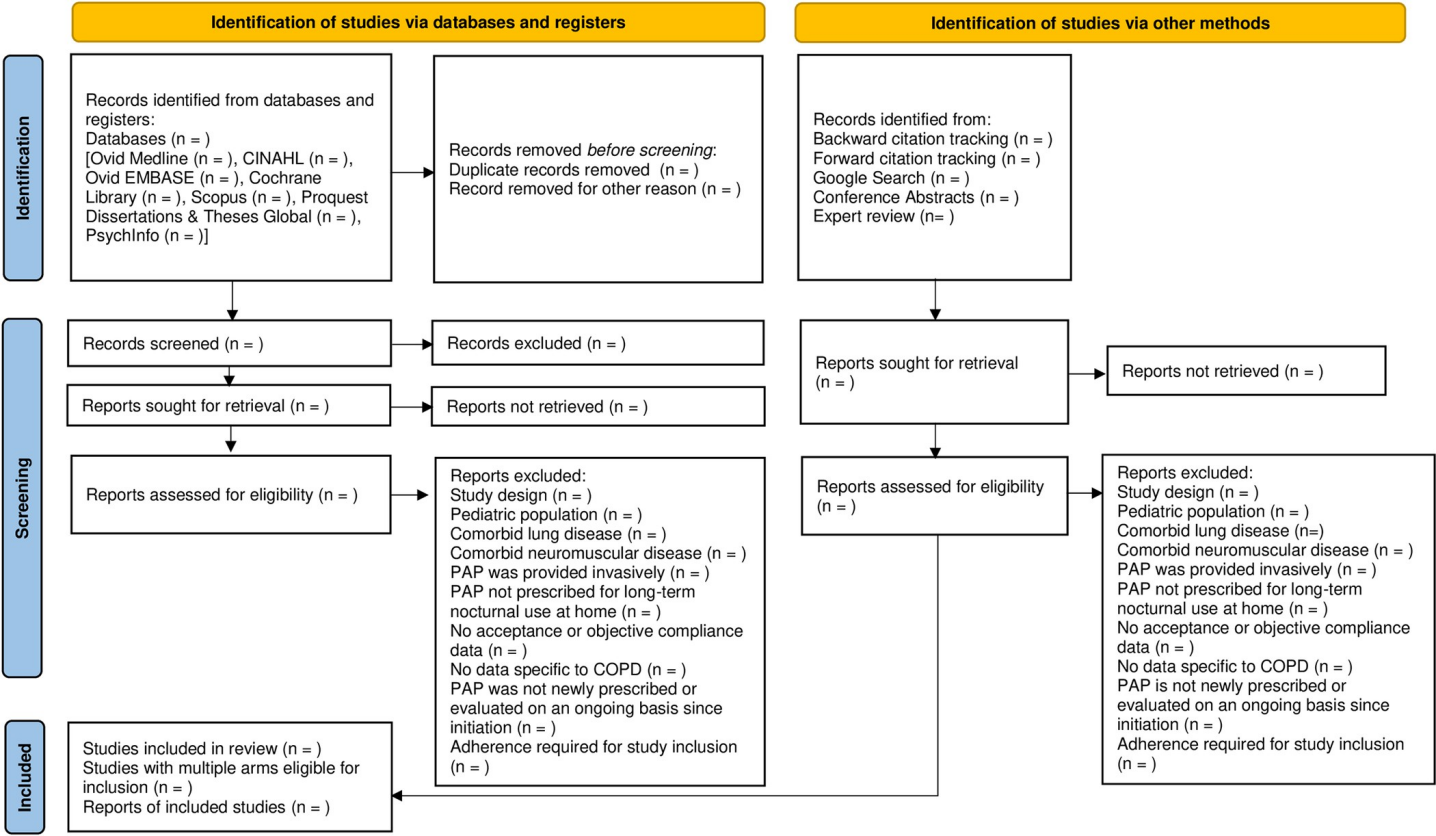
Anticipated flowchart of the included and excluded studies for the systematic review. The flowchart was developed following the following the Preferred Reporting Items for Systematic Reviews and Meta-analysis 2020 statement. CINAHL, Cumulative Index to Nursing and Allied Health Literature; EMBASE, Excerpta Medica Database; COPD, chronic obstructive pulmonary disease; PAP, positive airway pressure.

The methods for this systematic review are based upon the methodological frameworks outlined in the Cochrane Handbook for Systematic Reviews [26]. The results will be reported following the Preferred Reporting Items for Systematic Reviews and Meta-Analysis Protocols (PRISMA-P) 2020 statement [27]. For a PRISMA flow diagram, see Fig 1. The protocol was registered in the International Prospective Register of Systematic Reviews (PROSPERO; registration number CRD42021259262) after confirming that no similar systematic reviews were already registered. This study is exempt from ethics approval as this work is carried out on published documents.

### Eligibility criteria

#### Types of studies

Primary research studies reporting on randomized controlled trials or observational studies will be included; qualitative studies, letters to the editor, opinions and editorials will be excluded. For included studies, only arms of the trial in which PAP treatment was prescribed will be included. Study designs that have acceptance or adherence with PAP treatment as part of the inclusion or exclusion criteria will not be included in this review, unless the excluded participants are also characterized.

#### Types of participants/population

The review will include data analyses of adult patients (age ≥18 years) with COPD who are newly prescribed PAP treatment for long-term use at home for one of the following indications: chronic hypercarbic respiratory failure, OSA, central sleep apnea or sleep related hypoventilation. If a study population includes patients with chronic lung diseases where <80% of the patients have COPD, the data for the COPD population will be included only if it is reported separately from the other populations. Studies where all the participants have COPD, however, 20% or more of participants have neuromuscular disease, including diaphragmatic paralysis, or significant lung disease in addition to COPD (e.g., interstitial lung disease), will be excluded in order to decrease the heterogeneity in the study population. Studies will be excluded if they report on PAP treatment for acute respiratory failure in isolation or on patients treated during hospitalization with no intent to discharge on PAP treatment.

#### Exposures

PAP treatment will be defined by the provision of noninvasive PAP therapy through a nasal or face mask. CPAP is inclusive of fixed pressure or automatic titrating modalities. NIV is inclusive of bilevel positive airway pressure (BPAP), volume assured pressure support (VAPS) or adaptive servoventilation (ASV). Studies assessing mechanical ventilation delivered through an invasive interface such as a tracheostomy will be excluded. For studies that have multiple arms that meet the inclusion criteria, each arm will be summarized as a separate study population for the primary outcomes. Sham PAP will not be incorporated into the primary analysis of acceptance and adherence, as a lower adherence is seen with sham PAP in crossover trials [28]; however, given that sham PAP treatment has also been shown to be associated with lower adherence on CPAP therapy after transition to therapeutic treatment in the general population [28], it will be included in the secondary aim which is to summarize variables that may be associated with acceptance or adherence if there is sufficient data in the study population. During the systematic review, additional exposure variables associated with acceptance or adherence to PAP treatment in multivariate analysis will be collected and summarized. These additional variables will be organized within the World Health Organization framework [23] with two additional categories: research-specific factors (e.g., whether PAP equipment was provided to participants, co-interventions in the study design) and “Other”.

#### Comparator

Not applicable to this systematic review design.

#### Outcomes

**Fig 2:**
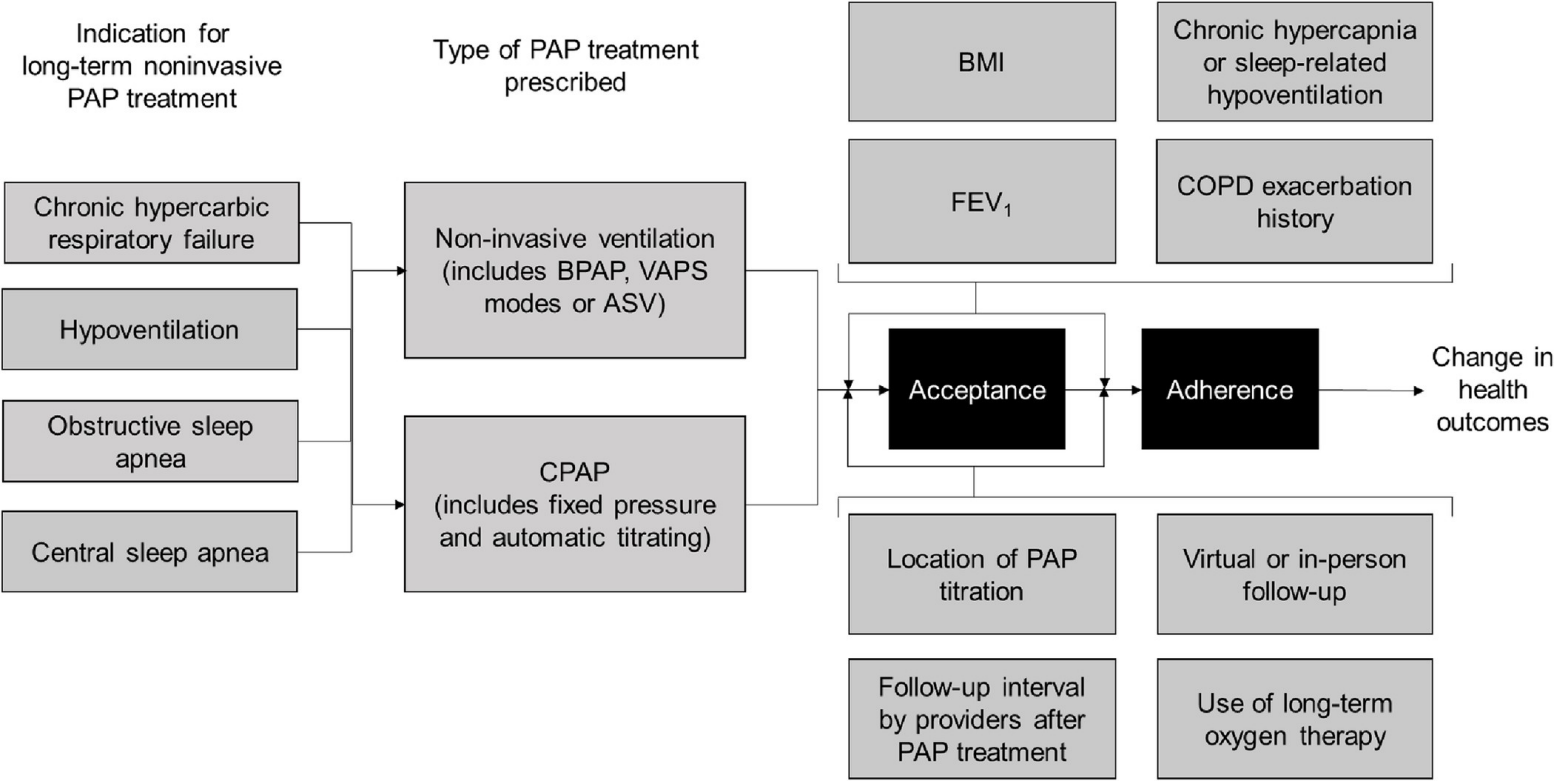
Logic model for summarizing the literature on PAP therapy acceptance and adherence in patients with COPD. Black boxes represent the primary outcomes of this study. Grey boxes represent prespecified clinically important subgroups that may explain some of the heterogeneity in the primary outcomes. White boxes will not be examined through this review. PAP, positive airway pressure; BPAP, bilevel positive airway pressure; VAPS, volume-assured pressure support; ASV, adaptive servoventilation; CPAP, continuous positive airway pressure; COPD, chronic obstructive pulmonary disease; BMI, body mass index; FEV1, forced expiratory volume in one second.

Primary outcomes will be acceptance of and adherence to long-term PAP treatment. Acceptance will be defined as dichotomous (i.e., had use of therapy irrespective of adherence compared to no use of therapy) within a given time period not exceeding 1 year from the therapy being recommended. Adherence will be determined from machine download data with a minimum of 7 days of follow-up. For studies that only report follow-up after a brief period of use, the adherence measured will be considered a proxy of long-term adherence given available evidence to suggest this in patients with OSA [29–31]. A sensitivity analysis will be conducted for adherence data collected within 1 month of initiating therapy in order to assess whether short term use is a proxy of long-term adherence in the included studies. Adherence will be defined as both dichotomous (e.g., >4 hours use per night for >70% of nights or >4 hours use per night for 5/7 days) and continuous (e.g., mean hours of use per night) where outcome measurement allows. Studies that only report adherence using subjective measures will be excluded. For a logic model, please see Fig 2. The secondary aim is to summarize known variables that are associated with either acceptance or adherence in this population.

#### Information sources

An experienced librarian and information specialist collaborated to design a comprehensive search strategy with terms for COPD, PAP treatment and adherence or acceptance. The search strategy (see S1 Appendix) was developed for Ovid Medline. This search will be translated into search strategies for CINAHL (EBSCOhost), Ovid EMBASE, the Wiley Cochrane Library (Cochrane Database of Systematic Reviews and Cochrane Central Register of Controlled Trials (CENTRAL)), Scopus, Proquest Dissertations & Theses Global and APA PsychInfo using the concepts of “obstructive airways disease” (e.g., COPD), “acceptance” or “compliance” or “adherence”, and “noninvasive positive pressure ventilation”. The search strategy will contain controlled vocabulary (e.g., Medical Subject Headings (MeSH)) and text words searches. There will be no restrictions on date of publication, publication status or language of publication; translation services will be employed to determine relevance of non-English manuscripts.

Grey literature will be searched including citation lists from the included studies. Corresponding authors of more than one included study will be contacted regarding unpublished studies or relevant literature that may have been missed. The publication lists for the last authors on the included studies will be reviewed. Indexes of conference abstracts will be hand searched from the American Thoracic Society, Canadian Thoracic Society, and European Respiratory Society, American College of Chest Physicians, Associated Professional Sleep Societies (joint venture by the American Academy of Sleep Medicine and the Sleep Research Society), Canadian Sleep Society, and the World Sleep Society from 2018–2023. The first 10 pages of a Google Scholar search will also be reviewed for relevant studies.

### Study records

#### Data management

The results of the searches will be imported into the Covidence software program and duplicates removed (Covidence systematic review software, Veritas Health Innovation, Melbourne, Australia). Where possible, statistical analyses will be completed using R statistical software (R Core Team, 2022) or Review Manager Software (RevMan, version 5.4, The Cochrane Collaboration, 2020).

#### Selection process

Two study investigators (CRL/LEM) will independently screen titles and abstracts identified by the search strategy for inclusion. If a large number of abstracts (>1000) are identified using the proposed search strategy, one study investigator will exclude obviously irrelevant studies based on title alone. Articles that are considered for inclusion by at least one author will be included in the full text review with subsequent documentation of exclusion criteria. In this phase, disagreements will be resolved by consensus or third-party involvement if consensus is not reached to establish the final list of studies to be included.

#### Data extraction

Data from included studies will be extracted by one of two reviewers (CRL, LEM, SWK) using a pre-designed data extraction form. The data extracted will then be independently verified for accuracy and completeness by the second author. Disagreements will be resolved by consensus through discussion or by adjudication involving a third reviewer. If relevant information is suspected to have been collected but not reported, attempts will be made to contact authors for clarification and data. See Data Extraction Form in Table 1.

**Table 1:** Data extraction form.

Study Characteristics	Extracted Data
General Information	First author, publication year, country/continent/multinational, source of funding, study period, study aims and research question/outcomes measured
Study Population	*General characteristics*: mean/median age, age range, sex, ethnicity, body mass index (BMI) *COPD disease characteristics*: method of defining COPD, forced expiratory volume in one second (FEV_1_), forced vital capacity (FVC), FEV1/FVC, diffusing capacity, arterial partial pressure of carbon dioxide (PaCO_2_), proportion with baseline PaCO_2_ >45 mmHg, use of home oxygen, exacerbation history, health-related quality of life *Details of PAP treatment*: indication for PAP treatment, Epworth Sleepiness Scale score, details of sleep diagnostic testing and results (type, AHI or surrogate measure, oxygen desaturation index, time spent with an oxygen saturation <90%, nadir oxygen saturation, improvement in PaCO_2_ or surrogate). *Details of PAP treatment*: including modality, settings, titration procedure or targets, mask interface type, follow-up method, frequency and duration Other Exposures: Variables associated with the primary outcome in multivariate analysis categorized as factors related to PAP indication, PAP treatment delivery or support, social and economic factors, COPD-specific variables, patient-related factors, health system and societal factors, research-specific factors or other
Study Design	Location of recruitment, patient enrollment process, sample size, inclusion and exclusion criteria, history of PAP use prior to study enrollment, how equipment for PAP was accessed (e.g. provided through the study or through insurance etc.), co-interventions, statistical methods used
Primary Outcome Measures	Acceptance, adherence
Authors Conclusions	Conclusions as reported by the authors
Gaps and Limitations Identified by Authors	Key limitations of the study as reported by the authors.

#### Quality assessment

Two reviewers will independently complete the risk or bias/quality assessment for each included study. For randomized control trials, the Cochrane Risk of Bias tool version 2.0 will be utilized. For case-control, cohort and cross-sectional studies, the Newcastle-Ottawa Scale will be used. A summary of the quality assessment will be reported in risk of bias graphs. Disagreements in risk of bias assessments will be resolved through consensus, and if consensus cannot be reached, then using third-party adjudication.

### Data synthesis

For the primary outcomes, a narrative synthesis will be performed and both study characteristics and results will be summarized in evidence tables. Following this, the data will undergo quantitative synthesis. For studies on PAP treatment acceptance or adherence, a weighted proportion or weighted average will be generated. The standard error of the weighted average will be used to derive a confidence interval, in order to inform the precision of the summary estimate.

For acceptance, the weighted proportion will subsequently be analyzed by stratifying by the two most common indications for PAP treatment, OSA and chronic hypercarbic respiratory failure, if sufficient data are available. For each subgroup analysis, the summary statistic will be calculated using a random-effects generic inverse-variance meta-analysis. With this analysis, evidence of statistical heterogeneity will be assessed as a p value from the Chi^2^ statistic of <0.10. Statistical heterogeneity will be quantified using the I^2^ summary statistic with values of <25%, 25–75% and > 75% representing low, moderate, and high degrees of heterogeneity, respectively. If the odds of acceptance are significantly different based upon whether the indication for treatment was OSA or chronic hypercarbic respiratory failure, the two groups will be analyzed for subsequent causes of heterogeneity separately; otherwise, subgroup analyses for causes of heterogeneity will be conducted on the whole population. The prespecified clinical subgroups selected through consensus by the study team members are as follows:

- Presence of OSA or chronic hypercarbic respiratory failure as the indication for PAP treatment;
- Commonly reported physiologic or historical factors of body mass index, forced expiratory volume in one second (FEV_1_), awake hypercapnia or sleep related hypoventilation, COPD exacerbation history, long-term oxygen therapy;
- Type of PAP treatment prescribed with a focus on CPAP or NIV;
- Location of PAP titration (hospital/polysomnography vs home), follow-up interval, and whether follow-up included in person assessments or virtual assessments after the initial set-up.

For adherence, the outcome measure may be reported as a mean and standard deviation (SD) or a median with other measures of spread such as minimum, maximum or interquartile range (IQR) or a proportion of adherent patients, where appropriate. If sufficient data are available for analysis of the proportions of adherent patients, a meta-analysis will be performed according to the methodology for acceptance outlined above. For adherence, where the outcome data are reported as a mean and SD or a median with measures of spread, the following analytic techniques will additionally apply. For consistency in reporting into figures, studies with outcomes reported using a median for non-normal distribution will be transformed using the method by Wan *et al*. [32] and Hozo *et al*. [33] to estimate a mean and standard error. The summary effect estimate; however, will be obtained by applying a meta-analysis using a medians-based approach, rather than a transformational method [34]. The use of this analysis does not provide an estimate of between-study heterogeneity; therefore, between-study heterogeneity will be assessed by stratifying the analysis of the difference in medians or means. Initially, the analysis of the weighted averages will be stratified by indication, either OSA or chronic hypercarbic respiratory failure. If it is medians or a mixture of medians and means measured, the data will be synthesized for these subgroups using the quantile estimation method to compare the difference of medians [35]. If means and SD are available for all studies, the summary statistic of a weighted mean difference will be calculated using a random-effects generic inverse-variance meta-analysis as per in the aforementioned methodology. After the weighted mean or median difference between the two groups is calculated, a Z-statistic will be applied. If these summary effect estimates are significantly different, the data will be further analyzed by stratifying the remaining subgroups within each indication. If there is no difference between these two groups, then the data will be analyzed as a whole through stratified analysis along the aforementioned relevant clinical subgroups. Each subgroup analysis will be examined to see if heterogeneity of the primary outcomes is explained by differences in subgroups prespecified below when the Chi^2^ distributions are compared using the degrees of freedom for between groups. For each analysis, a funnel plot will be constructed to identify the presence of publication bias if there are more than 10 included studies [26]. A sensitivity analysis will be used to assess the robustness of the results given the inclusion of various study types.

The secondary aim of this study will be to summarize variables found in studies to be associated in multivariate analysis with acceptance or adherence that could explain heterogeneity in the primary outcomes beyond the categories that were prespecified by the study team. For these variables, a narrative synthesis will be initially performed. Attempts will be made to dichotomize variables where there are multiple comparators or definitions (e.g., varying definitions or labels of the exposure rural versus urban). Where this synthesis is not possible, separate groups will be reported. The variables identified will be allocated into prespecified subcategories as previously described:

- Factors associated with the indication for PAP treatment or variables associated with COPD;
- Factors associated with PAP treatment delivery or support;
- Social and economic factors;
- Patient-related factors;
- Health system/societal factors;
- Research-specific factors;
- Other factors.

Where possible, data for the secondary aim will undergo quantitative synthesis where the exposure and comparator have sufficient data that have been similarly defined (e.g., rural exposure compared to urban exposure). A similar statistical approach to the analysis of the secondary outcomes will be undertaken as compared to the primary outcomes, depending on whether means, medians or proportions are reported. A pooled odds ratio or relative risk or a mean difference will be used to generate summary estimates where there is sufficient outcome data. No subgroup analyses will be conducted on the secondary outcome. Where it is not possible to synthesize the data into a meta-analysis due to high heterogeneity (I^2^>75%), lack of similarity in the way the exposure and comparator have been defined, differences in the population or inability to compare study designs, effect direction plots or forest plots will be used to synthesize the secondary outcomes.

A formal method of documenting confidence in the cumulative estimate (such as GRADE) will not be incorporated as the utility of this systematic review will depend on the health care context.

### Timeline

We anticipate finishing the search, screening, data extraction and synthesis by May 2023. Prior to completing the data synthesis, the search will be updated to ensure that all relevant literature has been captured prior to finalizing the study results.

## Discussion

This review protocol is novel in that it frames a methodological approach synthesizing the acceptance of and adherence with a complex intervention for which there is not a good comparison group. The strengths of this study are the methodological rigour with a registered and published protocol including detailed reporting of the outcomes and anticipated synthesis of the available data. The study population is well defined, as is the intervention being assessed. The searches for relevant studies will be comprehensive to reduce publication bias. The synthesis of the acceptance and adherence of PAP treatment across indications and interventions will allow for a detailed summary of the evidence. The pre-specified statistical analysis plan will summarize the measures of adherence and acceptance without a comparator, and still explore clinically relevant subgroups within the study population as a cause of heterogeneity in the primary outcomes that would allow for clinically meaningful interpretation of the results. A strength of this study is the additional effort to summarize exposure variables associated with the primary outcomes that were not prespecified by the study team in an effort to highlight any areas for future work going forward to explore heterogeneity in the primary outcomes.

Despite significant effort to ensure a strong methodological approach to this review, several study limitations are anticipated. The literature will be focused on specific indications and subgroups of clinical interest such as chronic hypercarbic respiratory failure in patients with COPD. This may limit generalizability of the synthesis results to the broader COPD population or to certain types of PAP treatment and reduce the value of efforts to quantitatively synthesize these data. Patients with overlapping indications for treatment may be underrepresented in this data synthesis. The statistical analysis may be challenging due to the various measures through which the primary outcomes of the study will be reported. Secondary variables are anticipated to also have variable definitions, which may preclude meaningful synthesis of the data. In anticipation of this, the qualitative and quantitative syntheses were developed to be comprehensive.

## Conclusion

To our knowledge, this will be the first systematic review to summarize data on acceptance of and adherence to PAP treatment in patients with COPD. Overall, the use of PAP treatment comes with a unique set of challenges. Through this protocol work, a clear methodology for a comprehensive and transparent systematic review of the existing literature on PAP acceptance and adherence in patients with COPD has been outlined. The prespecified data synthesis accounts for anticipated challenges with variability in measurement and anticipated analytic challenges of summarizing success with a complex intervention in a heterogeneous study population with heterogeneous measures of the primary outcomes. This review will provide a framework for strong methodological rigour in the synthesis of data on PAP treatment acceptance and adherence in patients with COPD.

## Supporting information

S1 Checklist PRISMA-P (Preferred Reporting Items for Systematic review and Meta-Analysis Protocols) 2015 checklist: Recommended items to address in a systematic review protocol*. (PDF)

S1 Appendix Search strategy for OVID Medline. (DOCX)

## List of abbreviations

PAP: positive airway pressure
COPD: chronic obstructive pulmonary disease
PRISMA-P: Preferred Reporting Items for Systematic Reviews and Meta-Analysis Protocols
PRISMA: Preferred Reporting Items for Systematic Reviews and Meta-Analysis Protocols
CPAP: continuous positive airway pressure
NIV: noninvasive ventilation
OSA: obstructive sleep apnea
PROSPERO: International Prospective Register of Systematic Reviews
BPAP: bilevel positive airway pressure
VAPS: volume assured pressure support
ASV: adaptive servoventilation
CENTRAL: Cochrane Database of Systematic Reviews and Cochrane Central Register of Controlled Trials
MeSH: Medical Subject Headings
RevMan: Review Manager Software
FEV_1_: forced expiratory volume in one second
FVC: forced vital capacity
BMI: body mass index
PaCO_2_: partial pressure of carbon dioxide
AHI: apnea-hypopnea index
