# Supplementary material for: Acceptance of and adherence with long-term positive airway pressure treatment in adults with chronic obstructive pulmonary disease: A systematic review protocol
Source: PLoS One. 2023 Jul 3;18(7):e0287887. doi: 10.1371/journal.pone.0287887 (PMC10317229; doi:10.1371/journal.pone.0287887)
Supplement: S1 Appendix — (DOCX) [file pone.0287887.s002.docx]

**Appendix A: Search Strategy for OVID Medline**

Ovid MEDLINE ® ALL <1946-April 12, 2021>

| 1 | exp Lung Diseases, Obstructive/ or (emphysema.mp. or chronic* adj3 bronchitis*.mp. or obstruct* adj3 (pulmonary or lung* or airway* or airflow* or bronch* or respirat*).mp. or (copd or coad or cobd or aecb).mp. or exp Pulmonary Disease, Chronic Obstructive/ |  |
| --- | --- | --- |
| 2 | exp Respiratory Therapy/ or exp Continuous Positive Airway Pressure/ or exp Positive-Pressure Respiration/ or exp Respiration, Artificial/ or exp Non-Invasive Ventilation/ or exp Intermittent Positive-Pressure Breathing/ or exp Intermittent Positive-Pressure Ventilation/ or (nippv or nppv or niv or niav or peep or bipap or cpap or ippb or ippv or BPAP or AVAPS or iVAPS or VAPS or PCV or PC or autoPAP or APAP or ASV or autoSV) not acute).mp. or (positive* adj3 pressur* adj5 (ventilat* or respir* or breath* or airway* or continuous or biphasic)).tw. or ((night* or non-invasive or noninvasive or nocturnal or domestic* or domicil* or home) adj3 ventilat*).mp. |  |
| 3 | 1 and 2 |  |
| 4 | exp "Patient Acceptance of Health Care"/ or exp Patient Satisfaction/ |  |
| 5 | (((user* or patient* or treatment* or intervention* or therap*) adj3 (accept* or toleran* or “non adher” or nonadher* or adher* or complian* or refusal* or refuse or refusing or dropout* or termian* or abandon* or uptake)) or ("hours of use" or "length of use" or "frequency of use")).mp. or exp Psychology/ |  |
| 6 | exp "Treatment Adherence and Compliance"/ or exp Patient Compliance/ or exp Patient Dropouts/ or exp Patient Adherence |  |
| 7 | or/4-6 |  |
| 8 | 3 and 7 |  |
